# Study protocols of three parallel phase 1 trials combining radical radiotherapy with the PARP inhibitor olaparib

**DOI:** 10.1186/s12885-019-6121-3

**Published:** 2019-09-10

**Authors:** R. de Haan, E. van Werkhoven, M.M. van den Heuvel, H. M. U. Peulen, G. S. Sonke, P. Elkhuizen, M. W. M. van den Brekel, M. E. T. Tesselaar, C. Vens, J. H. M. Schellens, B. van Triest, M. Verheij

**Affiliations:** 1 Department of Radiation Oncology, The Netherlands Cancer Institute, Plesmanlaan 121, 1066 CX Amsterdam, The Netherlands; 2 Department of Biometrics, The Netherlands Cancer Institute, Plesmanlaan 121, 1066 CX Amsterdam, The Netherlands; 3 Department of Thoracic Oncology, The Netherlands Cancer Institute, Plesmanlaan 121, Amsterdam, 1066 CX The Netherlands; 4 Department of Medical Oncology, The Netherlands Cancer Institute, Plesmanlaan 121, 1066 CX Amsterdam, The Netherlands; 5 Department of Head and Neck Surgery and Oncology, The Netherlands Cancer Institute, Plesmanlaan 121, 1066 CX Amsterdam, The Netherlands; 6 Division of Cell Biology, The Netherlands Cancer Institute, Plesmanlaan 121, 1066 CX Amsterdam, The Netherlands; 7 Division of Pharmacology, The Netherlands Cancer Institute, Plesmanlaan 121, 1066 CX Amsterdam, The Netherlands; 8 Department of Pharmaceutical Sciences, Utrecht University, Utrecht, The Netherlands

**Keywords:** Radiotherapy, Radiosensitisation, Olaparib, PARP inhibitor, Phase 1, Dose escalation, TITE-CRM, Dose limiting toxicity

## Abstract

**Background:**

Poly (ADP-ribose) Polymerase (PARP) inhibitors are promising novel radiosensitisers. Pre-clinical models have demonstrated potent and tumour-specific radiosensitisation by PARP inhibitors. Olaparib is a PARP inhibitor with a favourable safety profile in comparison to clinically used radiosensitisers including cisplatin when used as single agent. However, data on safety, tolerability and efficacy of olaparib in combination with radiotherapy are limited.

**Methods:**

Olaparib is dose escalated in combination with radical (chemo-)radiotherapy regimens for non-small cell lung cancer (NSCLC), breast cancer and head and neck squamous cell carcinoma (HNSCC) in three parallel single institution phase 1 trials. All trials investigate a combination treatment of olaparib and radiotherapy, the NSCLC trial also investigates a triple combination of olaparib, radiotherapy and concurrent low dose cisplatin. The primary objective is to identify the maximum tolerated dose of olaparib in these combination treatments, defined as the dose closest to but not exceeding a 15% probability of dose limiting toxicity. Each trial has a separate dose limiting toxicity definition, taking into account incidence, duration and severity of expected toxicities without olaparib. Dose escalation is performed using a time-to-event continual reassessment method (TITE-CRM). TITE-CRM enables the incorporation of late onset toxicity until one year after treatment in the dose limiting toxicity definition while maintaining an acceptable trial duration. Olaparib treatment starts two days before radiotherapy and continues during weekends until two days after radiotherapy. Olaparib will also be given two weeks and one week before radiotherapy in the breast cancer trial and HNSCC trial respectively to allow for translational research. Toxicity is scored using common terminology criteria for adverse events (CTCAE) version 4.03. Blood samples, and tumour biopsies in the breast cancer trial, are collected for pharmacokinetic and pharmacodynamic analyses.

**Discussion:**

We designed three parallel phase 1 trials to assess the safety and tolerability of the PARP inhibitor olaparib in combination with radical (chemo-)radiotherapy treatment regimens. PARP inhibitors have the potential to improve outcomes in patients treated with radical (chemo-)radiotherapy, by achieving higher locoregional control rates and/or less treatment associated toxicity.

**Trial registration:**

ClinicalTrials.gov Identifiers: NCT01562210 (registered March 23, 2012), NCT02227082 (retrospectively registered August 27, 2014), NCT02229656 (registered September 1, 2014).

**Electronic supplementary material:**

The online version of this article (10.1186/s12885-019-6121-3) contains supplementary material, which is available to authorized users.

## Background

Radical radiotherapy is widely used in patients with advanced solid tumours to achieve long-term locoregional control, translating into improvements in disease free and overall survival. The addition of chemotherapy has improved these outcomes in a broad range of tumour types [1]. Nevertheless, further improvements are warranted given both the toxicities of such radical treatments and the incidence of locoregional recurrences, especially as curative treatment options for recurrences are often limited. Dose escalation of radiotherapy and/or chemotherapy, however, is often limited by normal tissue toxicity. In addition, most radiosensitisers need to be applied intravenously, which is unpractical and invasive. Oral therapy would overcome these limitations.

Tumour specific radiosensitisers are expected to enhance tumour control without concomitantly increasing normal tissue toxicity. Potential tumour-specific radiosensitisers that are very promising in pre-clinical models include Poly (ADP-Ribose) Polymerase (PARP) inhibitors [2–4]. In patients the tolerability of PARP inhibitors as single agent compares favourably to most chemotherapeutic agents including cisplatin, currently the most widely used radiosensitiser in clinic [5–8]. Despite these promising characteristics of PARP inhibitors, little is known about the clinical value of PARP inhibitors as radiosensitisers [3].

PARP inhibitors are thought to radiosensitise through several different mechanisms. The best studied mechanism is through the inhibition of PARP1-mediated repair of radiation induced DNA damage. Other studies highlight the PARP1 trapping activity of such inhibitors that could interfere with cellular replication [9]*.* Radiosensitisation is more pronounced in replicating cells [10–13] and in cells that are homologous recombination deficient [10, 14, 15]. As both these properties are found more frequently in tumour cells compared to surrounding normal tissue cells, radiosensitisation is expected to occur preferentially in tumour cells. A second potential mechanism of tumour-specific radiosensitisation is based on the increase of tumour blood flow. Vessel dilation by several PARP inhibitors including olaparib increases tumour perfusion [16–20]. This efficiently reduces the hypoxic, radioresistant fraction in the tumour rendering the overall tumour more sensitive to radiation. It may also improve drug delivery leading to chemosensitisation.

In clinic, several orally bioavailable PARP inhibitors are used as standard of care or being developed. No clinical data on any PARP inhibitor in combination with radiotherapy was available at the time of the design of our combination trials. To date several trials have been published that assessed the tolerability of the PARP inhibitor veliparib in combination with radiotherapy. Radiotherapy in these combination trials was delivered to the whole abdomen [21, 22], the brain [23–27], the pelvic area for locally advanced rectal cancer [28] and the chest wall and regional lymph node areas for inflammatory or locoregionally recurrent breast cancer [29]. Haematological toxicity impeded the determination of a tolerable dose of veliparib in a triple treatment combination with radiation and temozolomide for glioblastoma multiforme [25]. All other trials found an acceptable safety profile of the combination treatment and identified a maximum tolerated dose (MTD) varying from 50 mg bi-daily to 400 mg bi-daily. Dose limiting toxicities (DLTs) included nausea and vomiting [27, 28], radiation dermatitis [28] and fibrosis [29]. A recent study reports the results of a phase 1 trial that combines the PARP inhibitor olaparib with cetuximab and radiotherapy for locally advanced head and neck squamous cell carcinoma (HNSCC) [30]. The recommended phase 2 dose of olaparib in this combination treatment was established at 25 mg bi-daily in tablet formulation. Dose limiting toxicity included dermatitis and nausea and vomiting. Grade 3–4 radiation dermatitis and mucositis were common (38 and 69% respectively). Awaiting results, the PARADIGM-2 study and OLA-TMZ-RTE-01 trial investigate the safety and tolerability of olaparib and radiotherapy with and without temozolomide in glioblastoma [31, 32]. To the best of our knowledge, there are no other published trial results on the combination of radiotherapy with olaparib.

To assess the clinical tolerability and safety of olaparib in combination with radiotherapy, we initiated three phase 1 trials in parallel: in patients with non-small cell lung cancer (NSCLC), breast cancer and HNSCC. Table 1 provides the rationales for the PARP inhibitor combination with radiotherapy in these specific types of cancer. All trials are investigator-initiated and single institution (The Netherlands Cancer Institute, the Netherlands). Olaparib is dose escalated in combination with a fixed radical (chemo-)radiotherapy regime in all trials to identify the MTD of olaparib in that specific combination treatment.

**Table 1:** Rationales to combine PARP inhibitors with radical (chemo-)radiotherapy

Rationale	Locally advanced NSCLC	Breast cancer	Locally advanced HNSCC
Locoregional recurrence rate despite primary curative (concurrent chemo-) radiotherapy	± 30% after platinum-based CCRT [33–35]	After combination of systemic treatment, surgery and radiotherapy: - 19–28% in high risk locally advanced breast cancer [36, 37] - 20% in inflammatory breast cancer, and up to 38% in triple negative inflammatory breast cancer [38, 39]	± 20–50% after platinum-based or cetuximab based CCRT [40–42]
Clinical evidence of benefit of DNA damaging radiosensitisers	Absolute benefits of platinum-based CCRT versus SCRT in meta-analysis [33]: - 5-year OS + 4.5% - 5-year LR control + 6.1%	N.A.	Absolute benefits of CCRT versus RT alone in meta-analysis [43]: - 5-year OS + 6.5% - 5-year LR control + 13.5% for 5-FU/platinum-based CCRT
Indicators of potential tumour specific radiosensitisation			
Frequency of HR deficiency	- Somatic mutations in BRCA1/BRCA2 (±8%) and ATM genes (±5%) [44, 45] *(biomarkers with clinical evidence of PARP inhibitor efficacy in other tumour types* [46–49]*)* - Alterations in other FA genes (±6%) and other HR genes (±16%) [50] *(biomarkers with* in vitro *evidence of PARP inhibitor efficacy* [51, 52]*)* See Additional file 2: Table S1.	- 5% of all breast cancer patients with germline BRCA 1 or BRCA2 mutations [53] *(biomarkers with clinical proof of benefit of olaparib and talazoparib monotherapy in phase 3 double blind randomized control trials* [54, 55] - Up to 20–25% of all breast cancer patients (40–70% in triple negative breast cancer subtype) with HR deficient / BRCA-like tumours^*#*^ *(biomarkers with clinical evidence of benefit of treatment with PARP inhibitors and/or DSB inducing agents*^*#*^*)* See Additional file 2: Table S2.	19–25% of all HNSCC patients with FA/HR gene alterations [50, 56, 57] *(biomarkers with* in vitro *evidence of PARP inhibitor efficacy* [51, 52] *and limited evidence of clinical benefit of high cumulative cisplatin dose in CCRT* [56]*)* See Additional file 2: Table S3.
Clinical benefit of hypoxia modifying strategies	N.A.	N.A.	In meta-analysis LR control + 8% versus RT alone, with a number needed to treat of 13 [58]

Several biomarkers for homologous recombination deficiency exist with different levels of evidence of PARP inhibitor efficacy. The table lists reported frequencies in order of level of evidence for PARP efficacy. CCRT = concurrent chemoradiotherapy, *SCRT* Sequential chemoradiotherapy, *RT* Radiotherapy, *OS* Overall survival, *LR* Locoregional, *5-FU* 5-fluoro-uracil, *HR* Homologous recombination, *BRCA1* BRCA1, DNA repair associated, *BRCA2* BRCA2, DNA repair associated, *FA* Fanconi anemia, *ATM* Ataxia telangiectasia mutated, *NA* Not applicable, # references in Additional file 2: Table S2

## Methods

### Inclusion criteria

The **NSCLC trial** includes patients in two parallel dose escalating arms. Patients can be included in the concurrent *chemoradiotherapy (CCRT) arm* if they have stage II/III inoperable disease without malignant pleural effusion. Patients can be included in the *(sequential chemo-)radiotherapy ((SC)RT) arm* if they have an indication for radical locoregional radiotherapy and, in case induction chemotherapy is given, if they have no disease progression after induction chemotherapy. This allows the inclusion of patients with oligometastatic disease with a good response to chemotherapy in the (SC) RT arm only [59].

The **breast cancer trial** includes patients with primary breast cancer or a local recurrence of breast cancer, including inflammatory breast cancer, which is inoperable and/or metastatic and that have an indication for breast irradiation. Patients with tumour involvement of the skin are included in a trial arm in which bolus material is used. Patients without tumour involvement of the skin are included in a parallel trial arm in which no bolus material is used. Concomitant use of tamoxifen or an aromatase inhibitor is allowed. Other systemic anti-cancer treatments are not allowed during the trial and up to four weeks after the last dose of olaparib.

The **HNSCC trial** includes patients with squamous cell carcinoma of the larynx (T2N0M0 or T1-2 N1-2bM0 or patients with locally advanced disease who will not receive CCRT) or oropharynx (T1-2 N1-2bM0 or T3 N0-2bM0 or patients with locally advanced disease who will not receive CCRT). Only oropharyngeal carcinoma patients with a HPV negative tumour status or oropharyngeal carcinoma patients with a history of smoking ≥ ten pack-years can be included (i.e. are at intermediate risk [60]).

Additional inclusion criteria in all three trials include: age higher than or equal to 18 years, a WHO performance status of 0–1 (a performance status of 2 is also allowed in the breast cancer trial only), a life expectancy of at least six months, adequate haematological, renal and hepatic functions, no concurrent active malignancy (other than non-melanoma skin cancer or carcinoma-in-situ), no prior radiotherapy to the involved region, no prior PARP inhibitor treatment, no concurrent CYP3A4 inhibitors of the following classes: azole antifungals, macrolide antibiotics, protease inhibitors and no gastrointestinal disorders that may interfere with absorption of the study drug.

### Study objectives and endpoints

The primary objective of all three trials is to identify the maximum tolerated dose (MTD) of olaparib in combination with radical radiotherapy. The highest dose level at which not more than 15% of patients experience dose limiting toxicity (DLT) or the highest reached dose level in the absence of DLT is defined as the MTD. The primary endpoint is the incidence of DLT. DLT is defined separately in each trial. We differentiate DLT definition between the acute toxicity phase (i.e. until three months after end of study treatment) and late toxicity phase (i.e. from three months until one year after end of study treatment) (Table 2). We aimed to define DLT so that it would not exceed a ‘baseline’ incidence of 10% after (chemo-)radiotherapy without olaparib.

**Table 2:** Definition of dose limiting toxicities in all three study protocols

	NSCLC trial	Breast cancer trial	HNSCC trial	All three trials
Acute toxicity phase	Non-hematological toxicity: • Radiation pneumonitis gr ≥ 3 • Any other non-hematological gr ≥ 3 toxicity (other than radiation esophagitis/dysphagia, radiation dermatitis, fatigue, nausea and vomiting, weight loss, anorexia and dehydration) • Gr ≥ 4 radiation esophagitis/dysphagia, radiation dermatitis, fatigue, nausea and vomiting, weight loss, anorexia and dehydration in the presence of maximal support/treatment. • Gr 3 radiation dermatitis present ≥ 9 weeks after end of treatment • Gr 2 cardiac or neurological toxicity Treatment discontinuation^#^: • Any radiotherapy discontinuation • Cisplatin cumulative discontinuation for > 20% of the total prescribed dose	Non-hematological toxicity: • Radiation dermatitis gr 4 except if this is associated with and at the localization of the ulcerative tumour • Radiation dermatitis gr 3 present ≥ 7 weeks after end of treatment • Pain related to dermatitis gr ≥ 3 present ≥ 7 weeks after end of treatment • Edema breast gr 3 in the presence of maximal support/treatment • Nausea and vomiting gr ≥ 3 in the presence of maximal support/treatment • Esophagitis gr ≥ 3 • Any other non-hematological toxicity gr ≥ 3 (except radiation dermatitis, pain, edema, nausea, vomiting, anorexia, weight loss and fatigue) Treatment discontinuation: • Any radiotherapy discontinuation due to toxicity attributable to radiotherapy, irrespective of the grade of toxicity • Cumulative discontinuation of radiotherapy for > 5 fractions due to toxicity attributable to olaparib, irrespective of the grade of toxicity	Non-hematological toxicity: • Gr ≥ 4 mucositis, dysphagia, radiation dermatitis, anorexia • Gr ≥ 3 hemorrhage, aspiration, trismus • Gr ≥ 3 radiation dermatitis present ≥ 8 weeks after end of treatment • Gr ≥ 3 nausea and/or vomiting in the presence of maximal support • Only in patients with oropharynx SCC: gr ≥ 3 larynx edema • Weight loss ≥ 20% of baseline weight Treatment discontinuation^#^: • Cumulative discontinuation of radiotherapy for > 3 fractions	Hematological toxicity: • Neutropenia gr 4 lasting for > 6 days • Neutropenic fever gr ≥ 3 • Thrombocytopenia gr 3 in the presence of bleeding; Thrombocytopenia gr ≥ 4 • Anemia gr 3 in the presence of blood transfusion dependency as judged by the PI; Anemia gr ≥ 4 Other: • Any other toxicity, which in the judgment of the Investigator is viewed as DLT Treatment discontinuation^#^: • Olaparib total discontinuation for > 20% of the total prescribed dose
Late toxicity phase	Non-hematological toxicity: • Gr ≥ 3 radiation pneumonitis, brachial plexopathy, esophageal stenosis, esophageal ulcer, esophageal necrosis, esophageal hemorrhage • Gr ≥ 2 myelitis, esophageal perforation, esophageal fistula	Non-hematological toxicity: • Fibrosis gr ≥ 3 outside the boost field AND if applicable outside the skin bolus field • Skin ulceration gr ≥ 2 except if this is persisting acute toxicity associated with and at the localisation of the ulcerative tumour • Radiation pneumonitis gr ≥ 3 • Esophagitis gr ≥ 3 • Brachial plexopathy gr ≥ 2 except if there was pre-existing brachial plexopathy or if the brachial plexopathy is tumour progression related	Non-hematological toxicity: • Gr ≥ 4 dysphagia, aspiration • Gr ≥ 3 hemorrhage, skin atrophy, trismus, osteoradionecrosis, radiation dermatitis, pneumonitis • Gr ≥ 2 fistula, myelitis • Gr ≥ 2 mucosal ulcer present ≥ 6 months after end of treatment • Fibrosis limiting joint or orifice movement (e.g. mouth) and/or limiting self-care ADL • Only in patients with oropharynx SCC: gr ≥ 3 larynx stenosis	Hematological toxicity: • Blood transfusion dependency as judged by the PI, unless the patient has progressive disease • Development of MDS/AML Other: • Any other toxicity, which in the judgment of the Investigator is viewed as DLT

The acute and late toxicity phases are defined as between start of treatment until three months after end of study treatment, and between three months until one year after end of study treatment respectively. All toxicities are graded according to CTCAE version 4.03 and are only considered a dose limiting toxicity (DLT) if they are assessed by the investigator as possibly, probably or definitely related to the combination of radiotherapy and olaparib. # Treatment discontinuation can be intermittent and/or continuous and is only considered a DLT if this is due to toxicity attributable to the combination study treatment, irrespective of the grade of toxicity. Gr = grade, ADL = activity of daily living, SCC = squamous cell carcinoma, MDS = myelodysplastic syndrome, AML = acute myeloid leukemia

Secondary objectives are:

- to describe the safety profile of olaparib in combination with radical radiotherapy
- to determine the pharmacokinetic profile of olaparib
- to assess the pharmacodynamic effects of olaparib
- to document preliminary evidence of anti-tumour effects.

Translational research within the trials has the exploratory objective to investigate the potential and feasibility of biomarkers in whole blood, serum and tumour biopsy samples as surrogate and/or predictive biomarkers for anti-tumour effects and/or treatment related toxicities.

### Study treatment

(Chemo-)radiation treatment details are summarised in Table 3. Radiation is delivered using IMRT (NSCLC, breast cancer) or VMAT-techniques (HNSCC). Following institutional guidelines, simultaneously integrated boost (SIB) and sequential boost techniques can be applied in the HNSCC trial. In the breast cancer trial, a boost to the macroscopic tumour in the breast is delivered using a SIB technique. Patients in the breast cancer trial with tumour involvement of the skin are irradiated using a skin bolus. Dose constraints to organs at risk follow institutional guidelines. Because of concerns of potentially increased risks of pneumonitis, for the first three patients in the NSCLC trial the maximal mean lung dose constraint is 16Gy instead of the maximal 20Gy accepted in institutional guidelines.

**Table 3:** (Chemo-)radiation treatment details

	Radiation target volumes	Radiation dose	Boost / Elective fields	Chemotherapy
NSCLC trial	Primary lung tumour and involved regional lymph nodes	66 Gy in 24 fractions	N.A.	Cisplatin 6 mg/m^2^ 1.5–2 h before radiation on every radiotherapy day^#^
Breast cancer trial	Whole breast and involved regional lymph node areas	46.69 Gy in 23 fractions	SIB of 14.49 Gy in 23 fractions to macroscopic tumour in breast; sequential boost of 10 Gy in 5 fractions to macroscopic lymph nodes (only if applicable)	N.A.
HNSCC trial	Primary tumour and regional metastatic lymph nodes; elective lymph node volumes based on site of primary tumour and stage	70 Gy in 35 fractions	Elective fields: 46 Gy in 23 fractions (sequential boost technique) or 54.25 Gy in 35 fractions (SIB technique)	N.A.

# in the CCRT arm only. Gy = Gray. N.A. = not applicable, SIB = simultaneous integrated boost

**Fig. 1:**
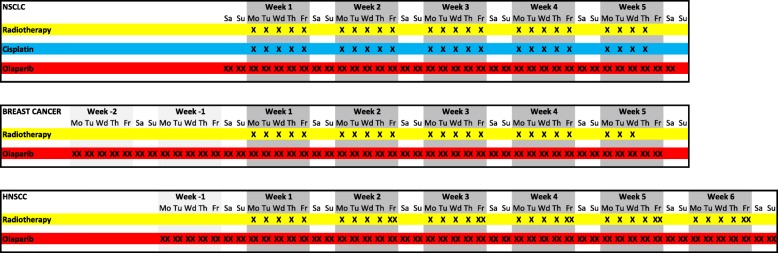
Treatment schedules in the NSCLC, breast cancer and HNSCC trials. Cisplatin is only given to patients in the CCRT arm. Radiotherapy in the HNSCC trial is delivered in five to six fractions per week in six weeks, the sixth fraction will be given on a weekday with an interval of at least six hours [61]

Olaparib is taken orally twice daily with a 12-h interval, starting two days before the first fraction of radiotherapy, continuing during weekends, until two days after the last fraction of radiotherapy. No specific time interval between radiation and olaparib intake is defined. To allow for translational research, olaparib is also given ahead of the combination treatment in two trials: two weeks before start of radiotherapy to take tumour biopsies in the breast cancer trial and one week before start of radiotherapy to acquire an extra MRI in the HNSCC trial. Figure 1 describes treatment schedules. Pre-defined dose levels are 25 mg once daily, 25 mg bi-daily, 50 mg bi-daily, 100 mg bi-daily, 200 mg bi-daily and 300 mg bi-daily. Olaparib is given in tablet formulation. Olaparib 25 mg once daily is a predefined dose de-escalation level, see Additional file 1 for details. The starting dose level is olaparib 50 mg bi-daily in the breast cancer trial arm without the use of a skin bolus and 25 mg bi-daily in all other trial arms. These are relatively low doses compared to the recommended monotherapy dose (300 mg bi-daily in tablet formulation [62]). We chose these as our preclinical data showed radiosensitisation at significantly lower olaparib concentrations than required for monotherapy efficacy [14]. Considering the available patient pharmacokinetic data [62] and dependence on the genetic tumour background one could expect radiosensitisation around 25 mg olaparib dose (tablet formulation) with an dose enhancement factor between 1.2 and 1.6 [14]. The starting dose level in the breast cancer trial arm without the use of a skin bolus is higher as the incidence, severity and duration of expected dose limiting toxicity such as dermatitis and mucositis is lower.

### Dose escalation design

The expected incidence, duration and severity of toxicities strongly depend on the use of a skin bolus (breast cancer trial) and on the use of concurrent chemotherapy (NSCLC trial). Therefore, olaparib is dose escalated separately in patients with and without the use of a skin bolus or concomitant chemotherapy. This results in two parallel study arms in the NSCLC trial and in the breast cancer trial, and only one trial arm in the HNSCC trial. As a consequence, the identified MTD can differ not only between trials but also between the two study arms within the same trial. Each trial arm accrues an estimated maximum of 36 patients evaluable for DLTs.

The NSCLC trial was launched first and started with a standard 3 + 3 design [63]. However, to be able to include late onset toxicity in the DLT definition, the dose escalation method was switched to a Time-to-Event Continual Reassessment Method (TITE-CRM [64, 65]). The breast cancer trial and HNSCC trial directly started with a TITE-CRM design. TITE-CRM is recommended for radiotherapy studies in several guidelines as late onset toxicity can be dose limiting [1, 66–68]. In short, TITE-CRM uses a dose level - toxicity model to identify the MTD, thereby weighting patients according to their time of follow-up. It allows the trial to continue with patient enrolment while previous patients are still being evaluated for late onset DLT. The first three patients in each trial arm are treated at the starting dose level. Thereafter, patients are assigned to a dose level using TITE-CRM and dose escalation rules. Upon enrolment of a new patient, TITE-CRM estimates the current MTD (see statistical analysis). New patients are assigned to the dose level that is closest to but not exceeding this current estimated MTD after applying two restrictive dose-escalation rules: 1) at least three patients have completed a minimal follow-up time of three months after end of treatment at the dose level below the assigned dose level, and 2) the assigned dose level may not increase more than one dose level between two consecutive patients. There is no restriction on the decrease in number of levels between consecutive patients.

### Assessments

Toxicity is assessed using the common terminology criteria for adverse events (CTCAE) version 4.03 at baseline, weekly during treatment, five times during the acute toxicity phase and at all follow-up visits during the late toxicity phase. The frequency and duration of follow-up visits in the late toxicity phase differs between trials: three monthly until two years after end of treatment (EOT) in the breast cancer trial, three monthly until one year after EOT and six monthly between one and five years after EOT in the NSCLC trial, and two monthly until one year after EOT and three monthly between one and two years after EOT in the HNSCC trial. Patients in all trials are followed until the last planned study follow-up visit or until disease progression, whichever occurs first. There is one exception, if disease progression occurs within the first year after EOT, toxicity assessments are completed until one year after EOT to be able to fully assess the DLT incidence. To evaluate cosmetic outcome in the breast cancer trial, breast appearance of the irradiated breast is scored according to Harvard criteria [69] at baseline and three-monthly until two years after EOT. Additionally, clinical photographs are taken at baseline, one week, three months and one year after EOT.

Treatment response is assessed using a tailored method in each trial: using a chest CT and FDG PET-CT scan six weeks after treatment in the NSCLC trial, using a prone breast-MRI three months after treatment in the breast cancer trial and a CT (only in case of larynx carcinoma) and MRI of the head and neck region three months after treatment in the HNSCC trial. Additional treatment response assessments at later time points are defined in the study protocols and follow institutional guidelines.

### Translational research

The primary purpose of translational research studies included in these trials is to investigate the biologically effective dose range of olaparib in combination with radiotherapy. It has been shown that much lower olaparib concentrations than those causing single agent cytotoxicity are required to be effective as radiosensitiser [14]. To investigate the biologically effective dose range of olaparib in patients, pharmacokinetic (PK) and adapted pharmacodynamic (PD) studies are conducted. Patient material is collected at several time points for these PK/PD studies. Plasma samples for PK analysis are taken on the day of the fourth radiation fraction (i.e. at near steady state olaparib levels [70]) at several time points in all trials: before olaparib intake and 0.5, 1, 2, 3, 4, 6, 8 and 12 h after intake. Blood samples are taken for PD analysis in all trials before start of treatment, several times during study treatment at three hours after olaparib intake and shortly before the next olaparib intake, and after end of treatment. An additional plasma sample is taken at each of these time points to assess PK-PD relationships. The collection of tumour biopsy material is restricted to the breast cancer trial and planned both before the start of treatment and at three hours after olaparib intake during the olaparib pre-treatment phase (week − 1 in Fig. 1).

Olaparib concentrations are measured in plasma and tumour samples using a validated high-performance liquid chromatography-tandem mass spectrometry (HPLC-MS/MS) method [71]. Pharmacodynamic analyses primarily focus on biological effective target inhibition in the context of radiation-induced responses, in this case radiation-induced PARylation. PAR levels are measured using our previously reported modified ‘REP-assay’ (radiation-enhanced-PAR-assay [72]). This ELISA-based PD assay includes an ex vivo irradiation step of intact cells to activate PARylation by PARP. The assay’s sensitivity to detect PARP inhibition was greatly increased by this modification and revealed inhibition of radiation-induced PARylation at low olaparib concentrations in a healthy volunteer study [72]. In our trials, the inhibition of radiation-induced PAR levels is assessed both in peripheral blood mononuclear cells from all trial patients, and in tumour biopsy material from breast cancer trial patients.

Another pharmacodynamic study aims to investigate the reported vasoactive properties of some PARP inhibiting compounds that alter tumour perfusion [16–20]. The associated reduction in tumour hypoxia and improvement in tumour drug delivery will be important determinants of response to radiotherapy and combined treatment. Tumour perfusion changes are therefore investigated by dynamic contrast enhanced MRIs in the HNSCC trial. MRI images are acquired in radiation mask before start of treatment and 1.5–2 h after olaparib intake during the olaparib pre-treatment phase (week − 1 in Fig. 1). Furthermore, PARP inhibitors may impact healthy tissue toxicity by interfering in the inflammatory responses induced by radiotherapy. Repeated serum samples are taken before, during and after treatment for analyses of changes in markers related to the radiation-induced inflammatory response, such as TGF-beta [73, 74]. Additionally the feasibility and potential value of candidate response biomarkers for the combination treatment of olaparib and radiotherapy is investigated. Given the increased radiosensitisation in DNA damage repair defected tumours [10, 14, 15] and in hypoxic tumours [17, 75], this includes biomarkers for DNA repair defects such as homologous recombination deficiency, and/or biomarkers for hypoxia. These additional genetic analyses are an optional part in the breast cancer trial and the HNSCC trial, of which patients are informed using a separate patient information file containing a separate form for consent.

### Treatment adherence and discontinuation

Adherence to daily oral olaparib intake is monitored closely. Actual drug intake is documented in a patient diary by patients themselves. A trained research nurse or radiation therapy technologist discusses olaparib intake and the patient diary completion before start of treatment and weekly until the end of treatment with each patient. Any discrepancies between returned tablets after end of treatment and documentation in the patient diary will be discussed with the patient.

Olaparib will be permanently discontinued in patients in whom a dose limiting toxicity occurs during treatment. Cisplatin, if given, will be discontinued until improvement in case of ≥ 30% reduction of glomerular filtration rate (GFR), a GFR < 50 ml/min or thrombocytopenia < 75 x 10E9/l. The decision to interrupt or change the radiotherapy regimen will be made on a case by case basis. In general radiotherapy will be continued in case of toxicity that can clearly be ascribed to olaparib or cisplatin, or in case only a limited number of radiotherapy fractions have to be delivered. In case of study treatment discontinuation after less than 80% of the planned cumulative olaparib dose, patients will have follow-up of all adverse events until resolution or until three months after the last dose of olaparib, whichever occurs first. In case of study treatment discontinuation after more than 80% of the planned cumulative olaparib dose, patients will undergo all planned study assessments.

### Serious adverse events

Serious adverse events (SAE) are defined according to the rules of Good Clinical Practice. All SAEs occurring until three months after end of study treatment must be reported. Thereafter, only possibly, probably or definitively related SAEs must be reported until the last study follow-up visit (see assessments). The above specified SAEs must be reported within one working day and are listed in an annually safety report.

### Data management and monitoring

Data will be collected on Case Report Forms and will be entered in database at the Department of Biometrics. Data cleaning will be performed following a study specific central data management plan. Source data verification will be performed by an independent Clinical Research Monitor. The study is considered to be high risk according to guidelines of the Dutch Federation of University Medical Centres. Therefore, 100% of all data will be monitored in the first three patients, and 100% of all primary data will be monitored in all subsequent patients. Drug-accountability will be performed by both the Clinical Research Monitor and Slotervaart Pharmacy. An auditing trial will not be performed routinely. All study results will be processed anonymously, identified by a patient verification code. All study files will be stored for 15 years.

### Statistical analysis

TITE-CRM uses a one parameter power model calculating the probability *P* of a DLT for a given dose *d* with the following formula: *P* (DLT/d) = d ^exp β^. The dose-toxicity parameter β is initially assumed to have a normal distribution with mean 0.0 and standard deviation of 1.16. Upon enrolment of a new patient, a new value of β is calculated based on a prior estimated DLT probability and the observed DLT rate in all already enrolled patients. The weight (w) of enrolled patients without DLT depends on their follow-up time according to a piecewise linear function which is w = 0 at start of treatment, w = 0.5 at three months after end of study treatment and w = 1 at one year after end of study treatment. This gives equal weight to the acute and late DLT periods. Patients experiencing a DLT are given the full weight (w = 1) independent of the time of DLT appearance and follow-up time. Subsequently *posterior DLT probabilities* with 90% Bayesian confidence intervals (“credible intervals”) are calculated for each dose level. The dose level that is closest to but not exceeding a 15% *posterior DLT probability* (i.e. the current estimated probability) is identified as the current estimated MTD. Once all patients in a trial arm completed their maximum DLT-observation period, this *posterior DLT probability* is the final estimate of the MTD. All calculations are performed at the statistical department of The Netherlands Cancer Institute using R software version 3.5.0 with package dfcrm version 0.2–2 [76].

## Discussion

The clinical development of radiosensitisers in a curative setting faces several important challenges [1, 66, 77]. First, concurrent chemotherapy regimens are often standard of care. Locally advanced NSCLC and locally advanced HNSCC are both treated with CCRT which is often cisplatin-based. Two clinical development strategies can be followed: one to compose a triple combination, or another to substitute the currently used chemotherapy by a novel radiosensitiser. The choice is based on the evaluation of expected efficacy, toxicity and feasibility. To the best of our knowledge, there are no preclinical data on a triple combination of radiation, cisplatin and a PARP inhibitor. Preclinical models have shown that PARP inhibitors not only sensitise tumours to radiation, but also to the well-established radiosensitiser cisplatin [78, 79]. Such triple treatments may therefore have strong anti-tumour effects. In clinic, nevertheless, other triple combinations did not always show superiority over the standard of care [40, 80, 81]. Also, triple combination treatments are often not well-tolerated [40, 80]. Mucositis and haematological toxicity are expected to be dose limiting in a triple combination of radiotherapy, cisplatin and a PARP inhibitor. Radiation induced mucositis is increased by cisplatin [33, 82, 83]. This could be aggravated by PARP inhibitors as both chemo- and radiosensitisation can occur especially in rapidly dividing tissue [10–13]). Haematological toxicity is shown to be dose limiting in a combination of olaparib and high dose cisplatin [84]. CCRT for HNSCC is given with a high dose of cisplatin and results in mucositis rates that are high and already close to dose limiting without a PARP inhibitor. We therefore reasoned that a triple combination in HNSCC would be too toxic. In the NSCLC trial, however, we decided to investigate the safety and tolerability of a triple combination treatment. Mucositis/esophagitis rates induced by cisplatin-based CCRT are substantially lower in NSCLC compared to HNSCC. Also, the lower cisplatin dose used in our CCRT schedule for NSCLC has a lower risk of haematological toxicity [85].

The second development strategy aims to substitute cisplatin by olaparib. Although a direct preclinical comparison of radiosensitisation potential between cisplatin and PARP inhibitors is lacking, reported radiation dose enhancement factors support a substitution strategy (i.e. around 1.2 for cisplatin [86] and 1.3–1.5 for PARP inhibitors [3, 14] in in vitro models). The substitution strategy is also supported by the favourable toxicity profile of PARP inhibitors. Systemic toxicity such as cisplatin induced nephrotoxicity will be avoided. Furthermore, olaparib has the advantages of being an oral drug over the intravenously injected cisplatin: non-invasive, more patient friendly and with less logistical issues. The challenge of the substitution strategy, however, concerns the identification of a large enough patient population for study inclusion. On the one hand, it is usually not considered acceptable to substitute a therapeutic agent for which level one clinical evidence of survival benefit exists (i.e. concurrent chemotherapy), with a therapeutic agent for which only preclinical evidence of radiosensitisation is available (i.e. in our trials olaparib). This possibly results in the ‘under treatment’ of patients. On the other hand, it is usually considered not justified to ‘over treat’ patients with metastatic disease with a radical radiotherapy regimen including a radiosensitiser that potentially even worsens toxicity. We defined our trial patient populations balancing between these ethical concerns. In the (SC) RT arm of the NSCLC trial we allow the inclusion of patients with oligometastatic disease, as their prognosis is comparable to that of patients with stage III disease in which the toxicity risks of radical radiotherapy are clinically accepted [59]. In the HNSCC trial we include patients with an intermediate risk for locoregional recurrence in whom standard of care treatment according to institutional policies is accelerated radiotherapy, only excluding HPV positive oropharyngeal tumours with a low risk of locoregional recurrence [60].

Another challenge in the clinical development of radiosensitisers concerns the strong dependency of toxicity profiles on radiation volumes and schedules [1, 66, 77]. The safety and tolerability of a radiosensitiser is therefore likely to depend on these same variables. The MTD for a given radiosensitiser can differ between primary tumour sites and radiotherapy schedules, as is shown for e.g. gemcitabine [1] and veliparib [21–25, 27–29]. Hence, we designed three parallel dose escalation trials. In addition, as the use of concurrent chemotherapy in NSCLC [33] or the use of a skin bolus in breast cancer [87–89] significantly increases toxicity, we designed parallel trial arms for separate dose escalation within the NSCLC trial and the breast cancer trial.

A final challenge in the clinical development of radiosensitisers concerns the DLT definition and evaluation period [1]. DLTs are typically defined as toxicities of grade three or more. In radical radiotherapy treatment regimens, however, certain grade three acute toxicities are common and considered clinically acceptable (e.g. dermatitis in the area where a skin bolus is used). In the case of such expected and accepted severe toxicities, DLTs can be defined as more severe than expected toxicity (e.g. grade four dermatitis) and/or as expected severe toxicity with a longer duration than expected (e.g. grade three dermatitis present ≥ seven weeks after end of treatment) [66]. A third strategy is to define a higher DLT probability as the MTD, taking a certain ‘baseline’ DLT probability with (chemo-)radiotherapy alone into account. We determined DLT definitions and acceptable DLT probabilities based on a structured literature review and expert opinions. The duration of the DLT evaluation period is also important, and can influence the final MTD dramatically. The MTD of veliparib in combination with radiotherapy to the chest wall and regional lymph node areas [29] was determined to be 200 mg bi-daily based on acute toxicity only. However, after considering the late onset toxicity that only started to occur one year after end of treatment, the final MTD was determined to be 50 mg bi-daily. Severe late onset toxicity was also found to appear six months after end of treatment in the recently published phase 1 trial combining olaparib with cetuximab and radiotherapy [30]. The MTD was based on acute toxicity only and determined to be 50 mg bi-daily. However, the recommended phase 2 dose was established at 25 mg bi-daily. Our trials have a DLT evaluation period until one year after end of treatment, as this time window captures most of the late onset toxicity such as late (consequential) oesophagus toxicity [90] and radiation pneumonitis [91].

In summary, we present three parallel single institutional phase 1 trials in which the PARP inhibitor olaparib is dose escalated in combination with radical (chemo-)radiotherapy in NSCLC, breast cancer and HNSCC patients to assess safety and tolerability of the combination treatments. Through the tumour-specific radiosensitisation of PARP inhibitors, these combination treatments have the potential to improve patient outcomes, by achieving higher locoregional control rates and/or less treatment associated toxicity.

## Additional files

Additional file 1: De-escalation dose level. Treatment schedules of the predefined dose de-escalation level of olaparib 25 mg once daily. (DOCX 69 kb)

Additional file 2: **Tables S1-S3**. Homologous recombination deficiency in NSCLC, breast cancer and HNSCC. The tables list reported gene mutation frequencies, if applicable BRCA-ness frequencies, and data showing preclinical and clinical PARP efficacy. (DOCX 147 kb)

## Data Availability

Not applicable.

## Abbreviations

(SC)RT: (sequential chemo-)radiotherapy
5-FU: 5-fluoro-uracil
ADL: activity of daily living
AML: acute myeloid leukemia
ATM: ataxia telangiectasia mutated
BRCA1: BRCA1, DNA repair associated
BRCA2: BRCA2, DNA repair associated
CCRT: concurrent chemoradiotherapy
CTCAE: common terminology criteria for adverse events
DLT: dose limiting toxicity
EOT: end of treatment
FA: fanconi anemia
GFR: glomerular filtration rate
gr: grade
Gy: Gray
HNSCC: head and neck squamous cell carcinoma
HPLC-MS/MS: high performance liquid chromatography-tandem mass spectrometry
HPV: human papillomavirus
HR: homologous recombination
IMRT: intensity modulated radiation therapy
LR: locoregional
MDS: myelodysplastic syndrome
METC: Medisch-Ethische Toetsingscommissie (medical ethics committee)
mg: milligram
MTD: maximum tolerated dose
NA: not applicable
NSCLC: non-small cell lung cancer
OS: overall survival
PARP: poly (ADP-ribose) polymerase
PBMCs: peripheral blood mononuclear cells
PD: pharmacodynamic
PK: pharmacokinetic
REP-assay: Radiation-Enhanced-PAR assay
RT: radiotherapy
SCC: squamous cell carcinoma
SIB: simultaneous integrated boost
TITE-CRM: time-to-event continual reassessment method
VMAT: volumetric modulated arc therapy
w: weight
