# Supplementary material for: Study protocols of three parallel phase 1 trials combining radical radiotherapy with the PARP inhibitor olaparib
Source: BMC Cancer. 2019 Sep 10;19:901. doi: 10.1186/s12885-019-6121-3 (PMC6734274; doi:10.1186/s12885-019-6121-3)
Supplement: Supplementary file 1 — De-escalation dose level. Treatment schedules of the predefined dose de-escalation level of olaparib 25 mg once daily. (DOCX 69 kb) [file 12885_2019_6121_MOESM1_ESM.docx]

**Treatment schedules of the predefined dose de-escalation level of olaparib 25mg once daily.**


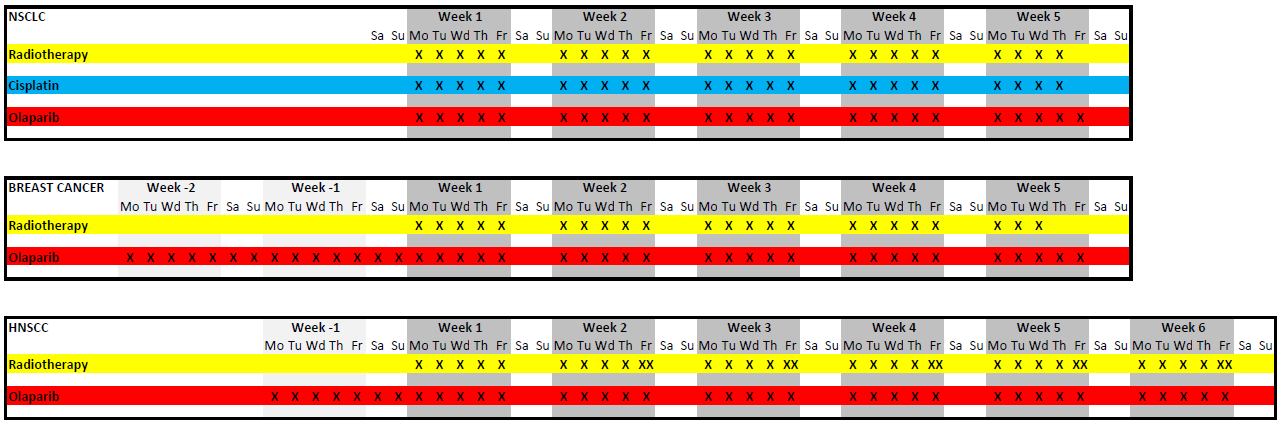


**Additional file 1: De-escalation dose level**. Cisplatin is only given to patients in the concurrent chemoradiotherapy arm of the non-small cell lung cancer (NSCLC) trial. Radiotherapy in the head and neck squamous cell carcinoma (HNSCC) trial is delivered in five to six fractions per week in six weeks, the sixth fraction will be given on a weekday with an interval of at least six hours (*Overgaard, 2003, Lancet*). Olaparib is taken orally once daily with a 24-hour interval 1.5-2 hours before radiation on radiotherapy days only. Olaparib is also given two weeks or one week ahead of the combination treatment in the breast cancer trial and HNSCC trial respectively to allow for translational research (i.e. tumour biopsies in the breast cancer trial and an MRI in the HNSCC trial).
