# Supplementary material for: Study protocols of three parallel phase 1 trials combining radical radiotherapy with the PARP inhibitor olaparib
Source: BMC Cancer. 2019 Sep 10;19:901. doi: 10.1186/s12885-019-6121-3 (PMC6734274; doi:10.1186/s12885-019-6121-3)
Supplement: Supplementary file 2 — Tables S1-S3. Homologous recombination deficiency in NSCLC, breast cancer and HNSCC. The tables list reported gene mutation frequencies, if applicable BRCA-ness frequencies, and data showing preclinical and clinical PARP efficacy. (DOCX 147 kb) [file 12885_2019_6121_MOESM2_ESM.docx]

**Homologous recombination deficiency in non-small cell lung cancer.**

| **Biomarker** | **Frequency** | **Evidence of therapeutic exploitability** |
| --- | --- | --- |
| FA/BRCA pathway | - HR gene alterations in 17% [1] incl. BRCA1/2 sm in 5-9% [2, 3] - FA gene alterations in 9% [1] incl. FANCM sm in 3.2% [3] - FANCF promotor methylation in 14% [4] | Clinical evidence of PARP inhibitor efficacy in ovarian cancer patients with somatic BRCA1/2 mutations from randomized controlled trials [5, 6]  In vitro sensitivity to PARP inhibitors in BRCA1-deficient NSCLC cell lines [7], in FANCM-deficient cell line [8] and in other FA/HR gene deficient cell lines [9, 10] |
| ATM/ATR | - ATM sm in 4.5-7%   (9% in adenocarcinoma; 4.2% in SCC) *^1,2^*   - ATR sm in 2.4% *^1^* | Clinical evidence of olaparib & rucaparib efficacy in patients with somatic mutations in ATM in non-randomized phase 2 studies [11, 12]  In vitro sensitivity to PARP inhibitors in ATM/ATR-deficient cell lines [9, 13, 14] |

**Supplemental Table 1.** Table lists reported gene alteration frequencies and data showing preclinical and clinical PARP efficacy. HR = homologous recombination, SCC = squamous cell carcinoma, sm = somatic mutations, ATM = ataxia telangiectasia mutated, ATR = ataxia telangiectasia and rad3 related, FA = fanconi anaemia, BRCA1 = breast cancer 1, early onset, BRCA2 = breast cancer 2, early onset , FANCM = Fanconi anemia complementation group M, FANCF = Fanconi anemia complementation group F. Level of evidence for tumor marker studies according to Simon et al. [15]

**Homologous recombination deficiency in breast cancer.**

| **Biomarker** | **Frequency** | **Clinical evidence of therapeutic exploitability** |
| --- | --- | --- |
| Germline BRCA mutations | 5% of all BC [16] | - Improved PFS (median PFS from 4.2 to 9 months with hazard ratio of 0.58) with olaparib monotherapy in metastatic breast cancer patients with a germline BRCA mutation in a phase 3 RCT, leading to FDA approval [17]. - Improved PFS (median PFS from 5.6 to 8.6 months with hazard ratio of 0.54) with talazoparib monotherapy in locally advanced or metastatic breast cancer patients with a germline BRCA mutation in a phase 3 RCT [18] - Promising objective response rates in breast cancer patients with germline BRCA mutations after niraparib and veliparib monotherapy [19] |
| Somatic BRCA mutations | Additional to germline BRCA mutations:   - 4% in all BC [20] - Enriched to 4-10% in basal like or triple negative BC [21, 22] | No clinical data in BC, however:   - Improved PFS (median PFS from 4.3 to 11.2 months with hazard ratio of 0.18) with olaparib maintenance treatment in platinum-sensitive recurrent serous ovarian cancer in a phase 2 RCT in a pre-planned sub-analysis of patients with a germline or somatic BRCA mutation, leading to EMA approval [6] - Improved PFS (median PFS from 5.4 to 16.6 months with hazard ratio of 0.23) with rucaparib maintenance treatment in platinum-sensitive recurrent ovarian cancer in a nested cohort in a phase 3 RCT, leading to FDA approval [5] |
| BRCA-ness  (based on genomic scars, gene expression profiles & functional assays) | Including germline and somatic BRCA mutations:   - 8-26% of all BC [23, 24] - Enriched to 41-78% in basal like BC / triple negative BC [22, 24-26] | - BRCA1-like DNA copy number profile is **predictive** for response (i.e. 5-fold lower change of recurrence) to **high dose** **chemotherapy** containing cross-linking drugs versus conventional chemotherapy regimens as adjuvant treatment in primary high risk BC patients [27-29]. Level of evidence: IB. - BRCA1-ness 77-gene expression profile is **predictive** for response (i.e. 2-4-fold higher pCR rates) to **veliparib/carboplatin**/ paclitaxel followed by AC versus paclitaxel followed by AC, as neo-adjuvant treatment in HER2 negative primary BC patients [26]. Level of evidence: IIB. - Low RAD51 score in tumor biopsy 24h after first chemotherapy cycle is **prognostic** for response (i.e. higher pCR rates: 33% versus 3%) to **anthracycline** based neo-adjuvant treatment in primary BC patients [24]. Level of evidence: IIIC. - SNP-based BRCA-high score prognostic for platinum-based neoadjuvant chemotherapy in BC [30, 31]; prognostic for response (i.e. hazard ratio for PFS 0.45) to niraparib in ovarian cancer [32], level of evidence: IIB. However, biomarker not predictive for clinical benefit of carboplatin in metastatic triple negative breast cancer patients, possibly due to selection of resistant clones by previous DNA-damaging treatments [22]. |

**Supplemental Table 2.** Table lists reported gene mutation frequencies, BRCA-ness frequencies and data showing preclinical and clinical PARP efficacy. HR = homologous recombination, BRCA1 = breast cancer 1, early onset, BRCA2 = breast cancer 2, early onset, BC = breast cancer, PFS = progression free survival, RCT = randomized control trial, FDA = Food and Drug Administration, EMA = European Medicines Agency, AC = doxorubicin and cyclophosphamide, SNP = single nucleotide polymorphism. Level of evidence for tumor marker studies according to Simon et al. [15]

**Homologous recombination deficiency in head and neck squamous cell carinoma.**

| **Biomarker** | **Frequency** | **Evidence of therapeutic exploitability** |
| --- | --- | --- |
| FA/HR gene variants | - FA/HR gene alterations in 19-25%   [1, 33, 34], enriched in laryngeal and pharyngeal SCC patients [34]   - FANCF promotor methylation in 15% [1, 4] | - Clinical evidence of PARP inhibitor efficacy in ovarian cancer patients with somatic BRCA1/2 mutations from randomized controlled trials [5, 6] - FA/HR gene alterations associated with worse prognosis (i.e. hazard ratio for death 2.6), but benefit from high cumulative cisplatin doses. Level of evidence: IIIC. [33] - In vitro sensitivity to PARP inhibitors in FA/HR gene deficient cell lines [9, 10] |

**Supplemental Table 3.** Table lists reported gene alteration frequencies and data showing preclinical and clinical PARP efficacy. HR = homologous recombination, FA = fanconi anemia, HR = homologous recombination, FANCF = fanconi anemia complementation group F. Level of evidence for tumor marker studies according to Simon et al. [15]

**References.**

1. Knijnenburg TA, Wang L, Zimmermann MT, Chambwe N, Gao GF, Cherniack AD, Fan H, Shen H, Way GP, Greene CS *et al*: Genomic and Molecular Landscape of DNA Damage Repair Deficiency across The Cancer Genome Atlas. Cell Rep 2018;23:1.

2. Campbell JD, Alexandrov A, Kim J, Wala J, Berger AH, Pedamallu CS, Shukla SA, Guo G, Brooks AN, Murray BA *et al*: Distinct patterns of somatic genome alterations in lung adenocarcinomas and squamous cell carcinomas. Nat Genet 2016;48:6.

3. Chae YK, Anker JF, Carneiro BA, Chandra S, Kaplan J, Kalyan A, Santa-Maria CA, Platanias LC, Giles FJ: Genomic landscape of DNA repair genes in cancer. Oncotarget 2016;7:17.

4. Marsit CJ, Liu M, Nelson HH, Posner M, Suzuki M, Kelsey KT: Inactivation of the Fanconi anemia/BRCA pathway in lung and oral cancers: implications for treatment and survival. Oncogene 2004;23:4.

5. Coleman RL, Oza AM, Lorusso D, Aghajanian C, Oaknin A, Dean A, Colombo N, Weberpals JI, Clamp A, Scambia G *et al*: Rucaparib maintenance treatment for recurrent ovarian carcinoma after response to platinum therapy (ARIEL3): a randomised, double-blind, placebo-controlled, phase 3 trial. Lancet 2017;390:10106.

6. Ledermann J, Harter P, Gourley C, Friedlander M, Vergote I, Rustin G, Scott CL, Meier W, Shapira-Frommer R, Safra T *et al*: Olaparib maintenance therapy in patients with platinum-sensitive relapsed serous ovarian cancer: a preplanned retrospective analysis of outcomes by BRCA status in a randomised phase 2 trial. Lancet Oncol 2014;15:8.

7. Paul I, Savage KI, Blayney JK, Lamers E, Gately K, Kerr K, Sheaff M, Arthur K, Richard DJ, Hamilton PW *et al*: PARP inhibition induces BAX/BAK-independent synthetic lethality of BRCA1-deficient non-small cell lung cancer. J Pathol 2011;224:4.

8. Shen Y, Rehman FL, Feng Y, Boshuizen J, Bajrami I, Elliott R, Wang B, Lord CJ, Post LE, Ashworth A: BMN 673, a novel and highly potent PARP1/2 inhibitor for the treatment of human cancers with DNA repair deficiency. Clin Cancer Res 2013;19:18.

9. McCabe N, Turner NC, Lord CJ, Kluzek K, Bialkowska A, Swift S, Giavara S, O'Connor MJ, Tutt AN, Zdzienicka MZ *et al*: Deficiency in the repair of DNA damage by homologous recombination and sensitivity to poly(ADP-ribose) polymerase inhibition. Cancer Res 2006;66:16.

10. Murai J, Huang SY, Das BB, Renaud A, Zhang Y, Doroshow JH, Ji J, Takeda S, Pommier Y: Trapping of PARP1 and PARP2 by Clinical PARP Inhibitors. Cancer Res 2012;72:21.

11. Mateo J, Carreira S, Sandhu S, Miranda S, Mossop H, Perez-Lopez R, Nava Rodrigues D, Robinson D, Omlin A, Tunariu N *et al*: DNA-Repair Defects and Olaparib in Metastatic Prostate Cancer. N Engl J Med 2015;373:18.

12. Swisher EM, Lin KK, Oza AM, Scott CL, Giordano H, Sun J, Konecny GE, Coleman RL, Tinker AV, O'Malley DM *et al*: Rucaparib in relapsed, platinum-sensitive high-grade ovarian carcinoma (ARIEL2 Part 1): an international, multicentre, open-label, phase 2 trial. Lancet Oncol 2017;18:1.

13. Lord CJ, McDonald S, Swift S, Turner NC, Ashworth A: A high-throughput RNA interference screen for DNA repair determinants of PARP inhibitor sensitivity. DNA Repair (Amst) 2008;7:12.

14. Turner NC, Lord CJ, Iorns E, Brough R, Swift S, Elliott R, Rayter S, Tutt AN, Ashworth A: A synthetic lethal siRNA screen identifying genes mediating sensitivity to a PARP inhibitor. EMBO J 2008;27:9.

15. Simon RM, Paik S, Hayes DF: Use of archived specimens in evaluation of prognostic and predictive biomarkers. J Natl Cancer Inst 2009;101:21.

16. Malone KE, Daling JR, Doody DR, Hsu L, Bernstein L, Coates RJ, Marchbanks PA, Simon MS, McDonald JA, Norman SA *et al*: Prevalence and predictors of BRCA1 and BRCA2 mutations in a population-based study of breast cancer in white and black American women ages 35 to 64 years. Cancer Res 2006;66:16.

17. Robson M, Im SA, Senkus E, Xu B, Domchek SM, Masuda N, Delaloge S, Li W, Tung N, Armstrong A *et al*: Olaparib for Metastatic Breast Cancer in Patients with a Germline BRCA Mutation. N Engl J Med 2017;377:6.

18. Litton J, Rugo HS, Ettl J, Hurvitz S, Gonçalves A, Lee KH, Fehrenbacher L, Yerushalmi R, Mina LA, Martin M *et al*: Abstract GS6-07: EMBRACA: A phase 3 trial comparing talazoparib, an oral PARP inhibitor, to physician's choice of therapy in patients with advanced breast cancer and a germline <em>BRCA</em> mutation. Cancer Res 2018;78:4 Supplement.

19. McCann KE, Hurvitz SA: Advances in the use of PARP inhibitor therapy for breast cancer. Drugs Context 2018;7.

20. Davies H, Glodzik D, Morganella S, Yates LR, Staaf J, Zou X, Ramakrishna M, Martin S, Boyault S, Sieuwerts AM *et al*: HRDetect is a predictor of BRCA1 and BRCA2 deficiency based on mutational signatures. Nat Med 2017;23:4.

21. Cancer Genome Atlas N: Comprehensive molecular portraits of human breast tumours. Nature 2012;490:7418.

22. Tutt A, Tovey H, Cheang MCU, Kernaghan S, Kilburn L, Gazinska P, Owen J, Abraham J, Barrett S, Barrett-Lee P *et al*: Carboplatin in BRCA1/2-mutated and triple-negative breast cancer BRCAness subgroups: the TNT Trial. Nat Med 2018;24:5.

23. Daemen A, Wolf DM, Korkola JE, Griffith OL, Frankum JR, Brough R, Jakkula LR, Wang NJ, Natrajan R, Reis-Filho JS *et al*: Cross-platform pathway-based analysis identifies markers of response to the PARP inhibitor olaparib. Breast Cancer Res Treat 2012;135:2.

24. Graeser M, McCarthy A, Lord CJ, Savage K, Hills M, Salter J, Orr N, Parton M, Smith IE, Reis-Filho JS *et al*: A marker of homologous recombination predicts pathologic complete response to neoadjuvant chemotherapy in primary breast cancer. Clin Cancer Res 2010;16:24.

25. Lips EH, Mulder L, Oonk A, van der Kolk LE, Hogervorst FB, Imholz AL, Wesseling J, Rodenhuis S, Nederlof PM: Triple-negative breast cancer: BRCAness and concordance of clinical features with BRCA1-mutation carriers. Br J Cancer 2013;108:10.

26. Severson TM, Wolf DM, Yau C, Peeters J, Wehkam D, Schouten PC, Chin SF, Majewski IJ, Michaut M, Bosma A *et al*: The BRCA1ness signature is associated significantly with response to PARP inhibitor treatment versus control in the I-SPY 2 randomized neoadjuvant setting. Breast Cancer Res 2017;19:1.

27. Schouten PC, Gluz O, Harbeck N, Mohrmann S, Diallo-Danebrock R, Pelz E, Kruizinga J, Velds A, Nieuwland M, Kerkhoven RM *et al*: BRCA1-like profile predicts benefit of tandem high dose epirubicin-cyclophospamide-thiotepa in high risk breast cancer patients randomized in the WSG-AM01 trial. Int J Cancer 2016;139:4.

28. Schouten PC, Marme F, Aulmann S, Sinn HP, van Essen HF, Ylstra B, Hauptmann M, Schneeweiss A, Linn SC: Breast cancers with a BRCA1-like DNA copy number profile recur less often than expected after high-dose alkylating chemotherapy. Clin Cancer Res 2015;21:4.

29. Vollebergh MA, Lips EH, Nederlof PM, Wessels LF, Schmidt MK, van Beers EH, Cornelissen S, Holtkamp M, Froklage FE, de Vries EG *et al*: An aCGH classifier derived from BRCA1-mutated breast cancer and benefit of high-dose platinum-based chemotherapy in HER2-negative breast cancer patients. Ann Oncol 2011;22:7.

30. Telli ML, Hellyer J, Audeh W, Jensen KC, Bose S, Timms KM, Gutin A, Abkevich V, Peterson RN, Neff C *et al*: Homologous recombination deficiency (HRD) status predicts response to standard neoadjuvant chemotherapy in patients with triple-negative or BRCA1/2 mutation-associated breast cancer. Breast Cancer Res Treat 2018;168:3.

31. Telli ML, Timms KM, Reid J, Hennessy B, Mills GB, Jensen KC, Szallasi Z, Barry WT, Winer EP, Tung NM *et al*: Homologous Recombination Deficiency (HRD) Score Predicts Response to Platinum-Containing Neoadjuvant Chemotherapy in Patients with Triple-Negative Breast Cancer. Clin Cancer Res 2016;22:15.

32. Mirza MR, Monk BJ, Herrstedt J, Oza AM, Mahner S, Redondo A, Fabbro M, Ledermann JA, Lorusso D, Vergote I *et al*: Niraparib Maintenance Therapy in Platinum-Sensitive, Recurrent Ovarian Cancer. N Engl J Med 2016;375:22.

33. Verhagen CVM, Vossen DM, Borgmann K, Hageman F, Grenman R, Verwijs-Janssen M, Mout L, Kluin RJC, Nieuwland M, Severson TM *et al*: Fanconi anemia and homologous recombination gene variants are associated with functional DNA repair defects in vitro and poor outcome in patients with advanced head and neck squamous cell carcinoma. Oncotarget 2018;9:26.

34. Vossen DM, Verhagen CVM, Verheij M, Wessels LFA, Vens C, van den Brekel MWM: Comparative genomic analysis of oral versus laryngeal and pharyngeal cancer. Oral Oncol 2018;81.
